# Nanofabrication on monocrystalline silicon through friction-induced selective etching of Si_3_N_4_ mask

**DOI:** 10.1186/1556-276X-9-241

**Published:** 2014-05-16

**Authors:** Jian Guo, Bingjun Yu, Xiaodong Wang, Linmao Qian

**Affiliations:** 1 Tribology Research Institute, National Traction Power Laboratory, Southwest Jiaotong University, Chengdu 610031, People's Republic of China

**Keywords:** Friction-induced selective etching, Si_3_N_4_ mask, Silicon

## Abstract

A new fabrication method is proposed to produce nanostructures on monocrystalline silicon based on the friction-induced selective etching of its Si_3_N_4_ mask. With low-pressure chemical vapor deposition (LPCVD) Si_3_N_4_ film as etching mask on Si(100) surface, the fabrication can be realized by nanoscratching on the Si_3_N_4_ mask and post-etching in hydrofluoric acid (HF) and potassium hydroxide (KOH) solution in sequence. Scanning Auger nanoprobe analysis indicated that the HF solution could selectively etch the scratched Si_3_N_4_ mask and then provide the gap for post-etching of silicon substrate in KOH solution. Experimental results suggested that the fabrication depth increased with the increase of the scratching load or KOH etching period. Because of the excellent masking ability of the Si_3_N_4_ film, the maximum fabrication depth of nanostructure on silicon can reach several microns. Compared to the traditional friction-induced selective etching technique, the present method can fabricate structures with lesser damage and deeper depths. Since the proposed method has been demonstrated to be a less destructive and flexible way to fabricate a large-area texture structure, it will provide new opportunities for Si-based nanofabrication.

## Background

Nanostructures of silicon have been widely used in micro/nanoelectromechanical systems (MEMS/NEMS) [1], photovoltaic devices [2-4], nanoimprint lithography template [5], and so on. As a typical nanofabrication method on silicon, photolithography technique involves complex systems and multiple steps [6,7]. Although it has a huge merit in mass production, photolithography is not suitable for flexible fabrication of micro-mold and prototype fabrication of microsystems [8]. Therefore, it remains essential to develop a simple and flexible nanofabrication technique to meet the requirements of nanoscience and nanotechnology.

Due to its simplicity, flexibility, and high resolution, scanning probe microscope (SPM)-based techniques have been demonstrated to hold great potential in fabricating nanostructures [9-14]. Among various SPM-based techniques of silicon, local anodic oxidation [13] and friction-induced selective etching [14] have attracted much attention from researchers. However, local anodic oxidation process strongly relies on the experimental parameters such as voltage, humidity, tip dwell time, and gaseous ambient environment [15]. Compared to local anodic oxidation technique, the friction-induced selective etching method has a more straightforward process and a lower requirement to environment. Without any additional facility, patterns can be easily fabricated by directly scratching a diamond tip on silicon substrate along the target trace and post-etching [16]. In this method, an affected layer is formed on the scratched area. Due to its resistance to alkaline solution, the affected layer can serve as an etching mask (defined as tribo-mask) for fabricating protrusive structures [17,18]. However, the etching selectivity of tribo-mask/Si(100) in KOH solution is low and uncontrollable [19]. When etching for a long time, the collapse may occur in the upper part of the structure [20]. Due to the restriction by the above factors, the maximum fabrication depth is generally less than 700 nm, which to some extent limits the application of the fabricated nanostructures [18]. To broaden the range of fabrication depth to micron scale, it is necessary to develop new fabrication methods with a high-quality mask. Since the etching selectivity of Si(100)/Si_3_N_4_ in KOH solution is about 2,600:1, the Si_3_N_4_ mask may be a good candidate by virtue of its excellent resistance to chemical attack [21].

In this paper, the friction-induced selective etching behavior of the Si_3_N_4_ mask on Si(100) surface was investigated. Effect of normal load and KOH etching period on fabrication depth was separately clarified. Based on the scanning Auger nanoprobe analysis, the fabrication mechanism of the proposed method was discussed. Finally, a large-area texture pattern with depth of several microns was attempted on Si(100) surface. The results may provide a simple, flexible, and less destructive way toward patterning a deep structure on silicon surface.

## Methods

Si(100) wafers coated with low-pressure chemical vapor deposition (LPCVD) Si_3_N_4_ films (Si/Si_3_N_4_) were purchased from Hefei Kejing Materials Technology, Hefei, China. X-ray photoelectron spectroscopy (XPS; XSAM800, Kratos, Manchester, UK) detection revealed that the deposited films were stoichiometric Si_3_N_4_. Scanning Auger nanoprobe (PHI 700, ULVAC-PHI, Inc., Kanagawa, Japan) detection indicated that the thickness of Si_3_N_4_ films was about 50 nm. Using an atomic force microscope (AFM; SPI3800N, Seiko, Tokyo, Japan), the root-mean-square (RMS) roughness of the Si/Si_3_N_4_ samples was measured to be 0.4 nm over a 2 μm × 2 μm area. The elastic modulus of the Si_3_N_4_ film was estimated to be 240 GPa by nanoindentation with a spherical diamond tip [22].

**Figure 1:**
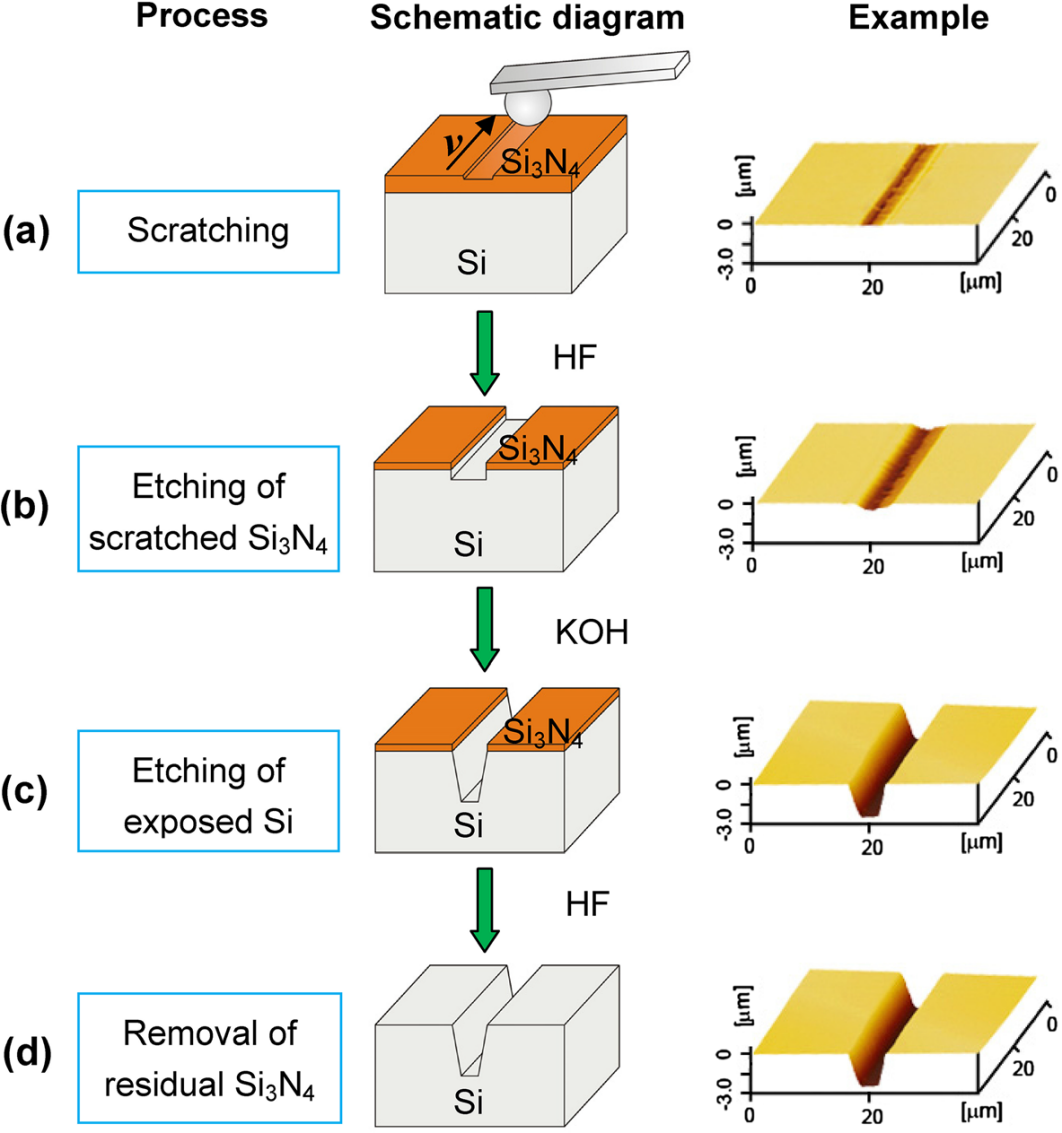
**Schematic illustration showing the fabrication process. (a)** Scratching a spherical diamond tip along the designed traces on the silicon sample coated with Si_3_N_4_ mask (Si/Si_3_N_4_). **(b)** Selective etching of the scratched Si_3_N_4_ mask in HF solution. **(c)** Selective etching of the exposed silicon in KOH solution. **(d)** Removing the residual Si_3_N_4_ mask by HF solution.

The whole fabrication process consisted of four steps, as shown in Figure 1. Firstly, scratching was performed on the Si/Si_3_N_4_ sample by a spherical diamond tip under a proper normal load (Figure 1a). Secondly, the Si_3_N_4_ film was selectively etched in hydrofluoric acid (HF) solution until the Si substrate was exposed on the scratched area (Figure 1b). Thirdly, with the mask effect of the residual Si_3_N_4_ film on the non-scratched area, the exposed Si was selectively etched in potassium hydroxide (KOH) solution (Figure 1c). Finally, the residual Si_3_N_4_ film was removed by HF etching (Figure 1d).

During the fabrication process, scratching was conducted on Si/Si_3_N_4_ samples by a nanoscratch tester (TI750, Hysitron Inc., Eden Prairie, MN, USA) using a spherical diamond tip with a nominal radius *R* of 1.5 μm. The large-area fabrication was realized by a self-developed microfabrication apparatus, on which the maximum fabrication area of 50 mm × 50 mm can be achieved [23]. During scratching process, the temperature was controlled at 22°C and the relative humidity ranged between 40% and 45%. In etching process, 2 wt.% HF solution was used for selective etching of the scratched Si/Si_3_N_4_ sample and removal of the residual Si_3_N_4_ layer; a mixture of 20 wt.% KOH solution and isopropyl alcohol (IPA) (volume ratio = 5:1) used for selective etching of the exposed silicon. The etching temperature was set to be 23 ± 1°C. All of the AFM images were scanned in vacuum by silicon nitride tips (MLCT, Veeco Instruments Inc., Plainview, NY, USA) with a spring constant *k* = 0.1 N/m. The morphology of large-area textured surface was observed by a scanning electron microscope (SEM; QUANTA200, FEI, Hillsboro, OR, USA). The contact angle of textured surface was tested by an optical contact angle measuring device (DSA-100, KIUSS, Hamburg, Germany).

## Results and discussion

### Friction-induced selective etching of Si_3_N_4_ mask in HF solution

**Figure 2:**
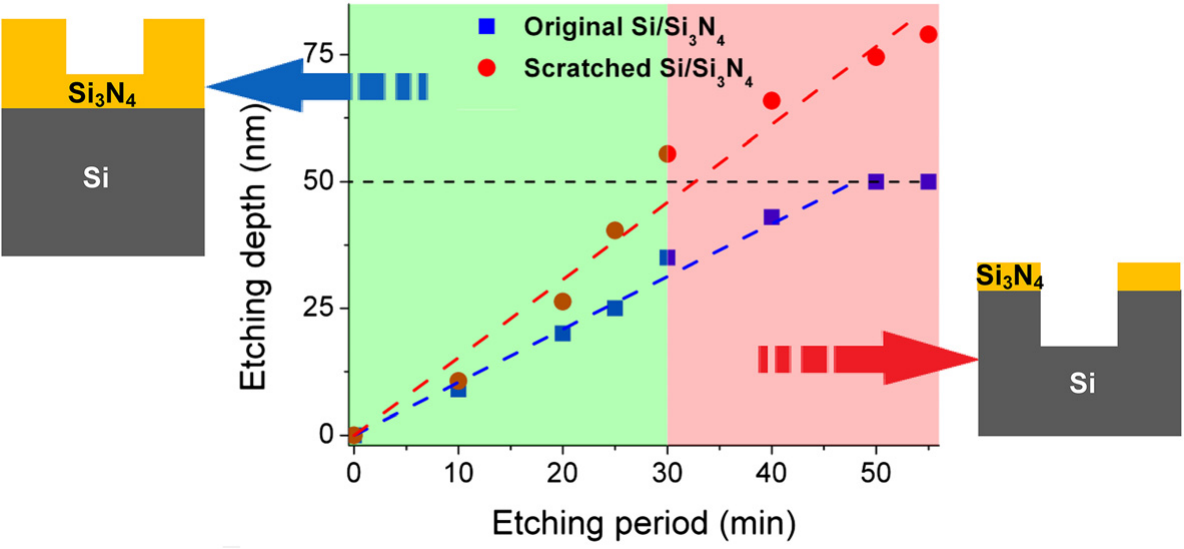
**Variation of etching depth of Si/Si**_**3**_**N**_**4 **_**sample with etching period in HF solution.** After etching for 30 min, Si was exposed on the scratched region while a residual Si_3_N_4_ mask of 15 nm in thickness was still covered on the original region.

In order to study the friction-induced selective etching behavior of the Si_3_N_4_ mask on Si(100) surface, nanoscratching was performed on a Si/Si_3_N_4_ sample under a normal load *F*_n_ of 3 mN. After scratching, plastic deformation occurred on the scratched area and a groove with residual depth of 1.1 nm was generated. After post-etching in HF solution for different periods, the thicknesses of residual Si_3_N_4_ mask layers on both the scratched area and the original area (non-scratched) were detected by a scanning Auger nanoprobe. As shown in Figure 2, the average etching rate on the original Si/Si_3_N_4_ surface was about 1.0 nm/min and on the scratched Si/Si_3_N_4_ surface was about 1.7 nm/min. The results indicated that HF solution could selectively etch the scratched Si/Si_3_N_4_ sample. After HF etching for 30 min, the etching depth of the scratched area was larger than 50 nm, while the thickness of the residual Si_3_N_4_ mask on the non-scratched area was 15 nm. At this moment, the Si_3_N_4_ mask on the scratched area was just etched off and the Si substrate was exposed on this area. This etching period was defined as the minimum etching period (*t*_min_) for fabrication of the Si/Si_3_N_4_ sample. After HF etching for 50 min, the residual Si_3_N_4_ mask on the non-scratched area was thoroughly removed. This etching period was defined as the maximum etching period (*t*_max_) for fabrication of the Si/Si_3_N_4_ sample. During fabrication process, the HF etching period was strictly controlled between *t*_min_ and *t*_max_. After selective etching of the scratched Si/Si_3_N_4_ sample in HF solution, the exposed Si can be selectively etched in KOH solution with the purpose of fabricating a deeper structure (as shown in Figure 1c). With the high etching selectivity of Si(100)/Si_3_N_4_ in KOH solution, the theoretical maximum fabrication depth can reach several microns.

### Effect of scratching load and KOH etching period on nanofabrication

**Figure 3:**
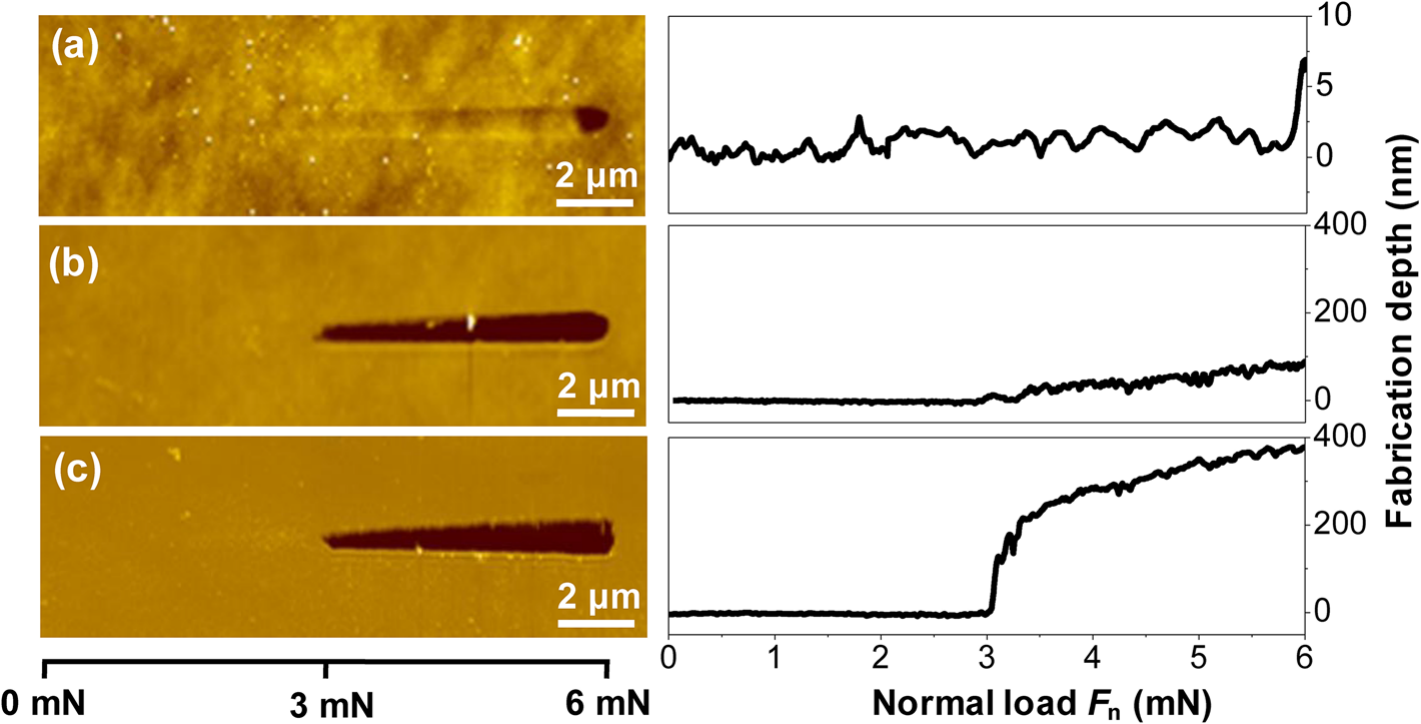
**Load effect on friction-induced selective etching of Si/Si**_**3**_**N**_**4 **_**sample. (a)** Scratching with progressive load from 0 to 6 mN. **(b)** Etching in HF solution for 30 min. **(c)** Further etching in KOH solution for 35 min.

As a friction-induced selective etching approach, both the scratching load and KOH etching period show strong effect on the nanofabrication of the Si/Si_3_N_4_ sample. To study the role of scratching load in fabrication, a scratch with a length of 15 μm was produced on the Si/Si_3_N_4_ surface under progressive load from 0 to 6 mN, as shown in Figure 3a. It was found that a slight wear began at about 3 mN. With the increase in normal load *F*_n_ from 3 to 6 mN, the wear depth gradually increased. After etching in HF solution for 30 min, part of the Si substrate was exposed on the scratched area and a groove was produced with depth ranging from 17 to 86 nm (the corresponding *F*_n_ ranging from 3 to 6 mN), as shown in Figure 3b. Finally, the sample was etched in KOH solution for 35 min, and a deeper groove was fabricated with depth varying from 130 to 385 nm (the corresponding *F*_n_ ranging from 3 to 6 mN), as shown in Figure 3c. The results indicated that the minimum *F*_n_ to cause selective etching of Si/Si_3_N_4_ was about 3 mN, under which the Hertzian contact pressure *P*_c_ was estimated to be about 18.4 GPa. With the increase in *F*_n_ from 3 to 6 mN, the corresponding selective etching depth gradually increased. It indicated that the minimum etching period decreased with the increase in the normal load.

**Figure 4:**
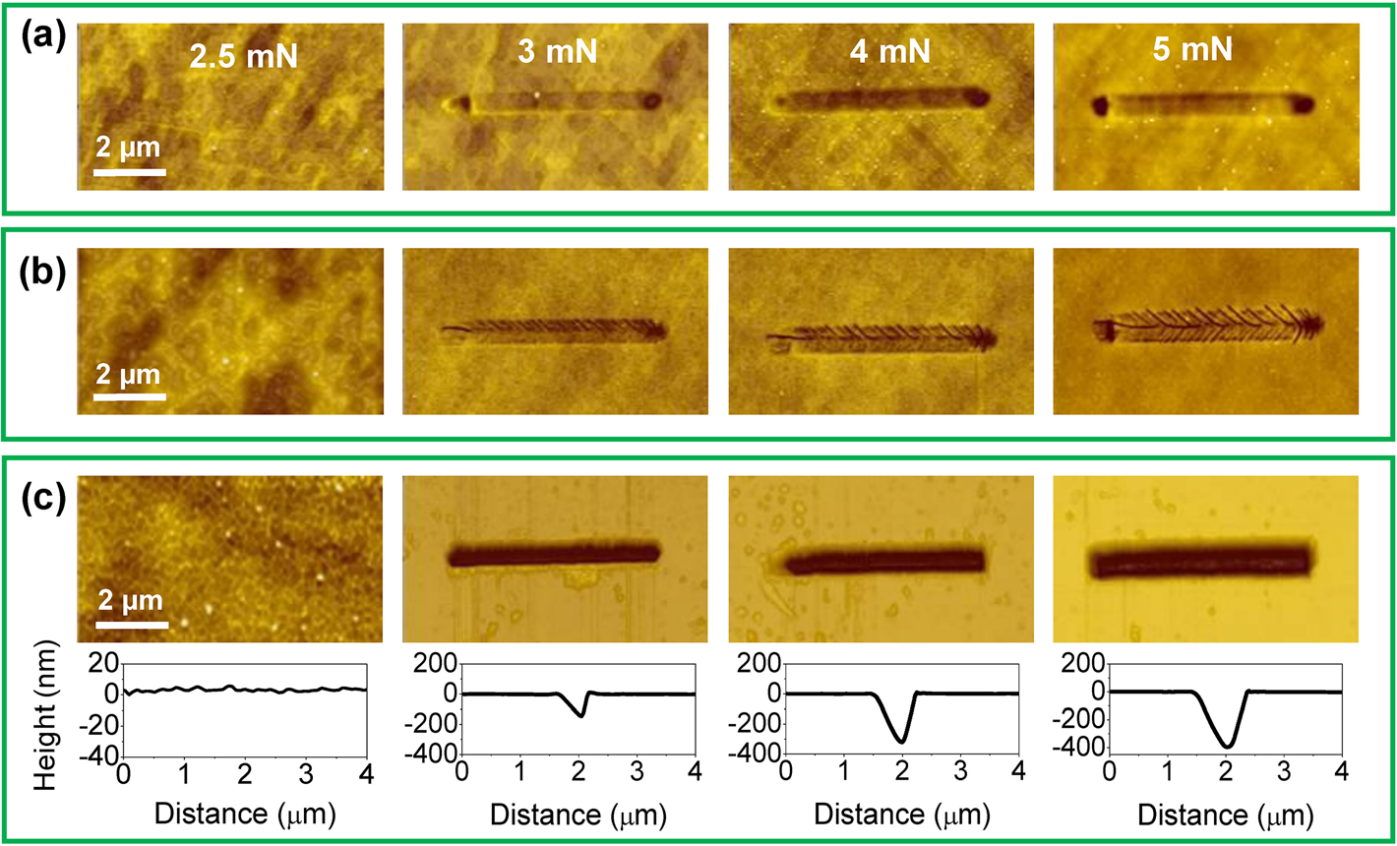
**Correlation of crack formation and selective etching of Si**_**3**_**N**_**4 **_**mask. (a)** Scratching under normal load *F*_n_ = 2.5, 3, 4 and 5 mN. **(b)** Crack formation after HF etching for 20 min. **(c)** Further etching in HF solution for 10 min and KOH solution for 35 min.

To further understand the load effect on the friction-induced selective etching of the Si/Si_3_N_4_ sample, the scratching tests were performed on a Si/Si_3_N_4_ sample under different constant loads. As shown in Figure 4a, no surface damage was observed on the scratched area when the normal load was 2.5 mN (*P*_c_ ≈ 17.5 GPa). Whereas, the depths of the grooves were 1.1, 2.1, and 3.8 nm under scratching loads of 3, 4, and 5 mN, respectively. After etching in HF solution for 20 min, some microcracks emerged on the groove area and the corresponding depths of grooves increased to 4.1, 5.2, and 10.4 nm, as shown in Figure 4b. After further etching in HF solution for 10 min and in KOH solution for 35 min, the depths of the grooves continually grew to 139, 320, and 398 nm (Figure 4c). Here, the selective etching of the Si/Si_3_N_4_ sample may be partly related to the formation of microcracks on the damaged area. Since the microcracks can accelerate the diffusion of the HF solution, the etching rate of the damaged Si/Si_3_N_4_ surface with microcracks is faster than that of the original Si/Si_3_N_4_ surface.

**Figure 5:**
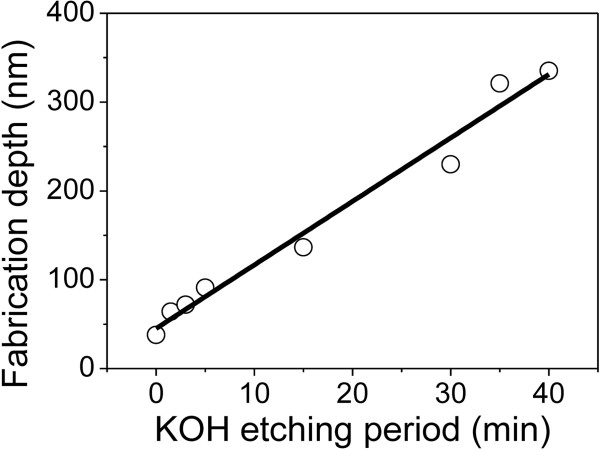
**Variation of fabrication depth of Si/Si**_**3**_**N**_**4 **_**sample with etching period in KOH solution.** Before KOH solution etching, the sample was scratched under *F*_n_ of 4 mN and then etched in HF solution for 30 min.

The effect of KOH etching period on nanofabrication was also studied. After scratching under *F*_n_ of 4 mN and etching in HF solution for 30 min, the Si substrate was exposed on the scratched area of the Si/Si_3_N_4_ sample. When the sample was further etched in KOH solution, the fabrication depth increased almost linearly with KOH etching period and the average etching rate was calculated as 7.1 nm/min, as shown in Figure 5. In summary, through the control of the scratching load and KOH etching period, it is convenient to fabricate a groove structure with a required depth.

### Fabrication of nanostructures on Si(100) surface

**Figure 6:**
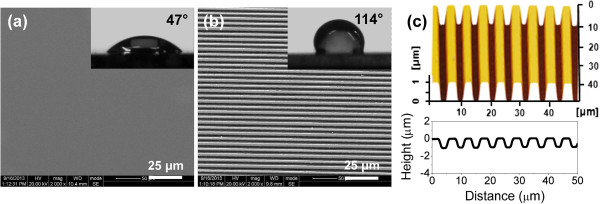
**Fabrication of large-area texture and contact angle tests. (a)** SEM image of the original Si(100) surface; the contact angle is tested at 47°. **(b)** SEM image of the Si(100) surface with texture, which was fabricated by nanoscratching under *F*_n_ = 50 mN and post-etching in HF solution for 30 min and KOH solution for 2 h in sequence; the contact angle is 114°. **(c)** AFM 3D-morphology of the partial texture in **(b)**.

Based on its large working area and fast scanning speed, the self-developed microfabrication apparatus provides a promising way for fabricating micro/nanometer-scale features on a large-size specimen. After scratching and post-etching, a large-area texture pattern was fabricated on a Si(100) surface, which consisted of 1,000 parallel grooves over a 5 mm × 5 mm area. As shown in Figure 6, the textured surface showed strong hydrophobicity, and the contact angle was tested to be 114° (Figure 6b), which was about 2.4 times that on the original Si(100) surface (Figure 6a). Such superhydrophobic textured surface has considerable technological potential in various applications [24-26].

**Figure 7:**
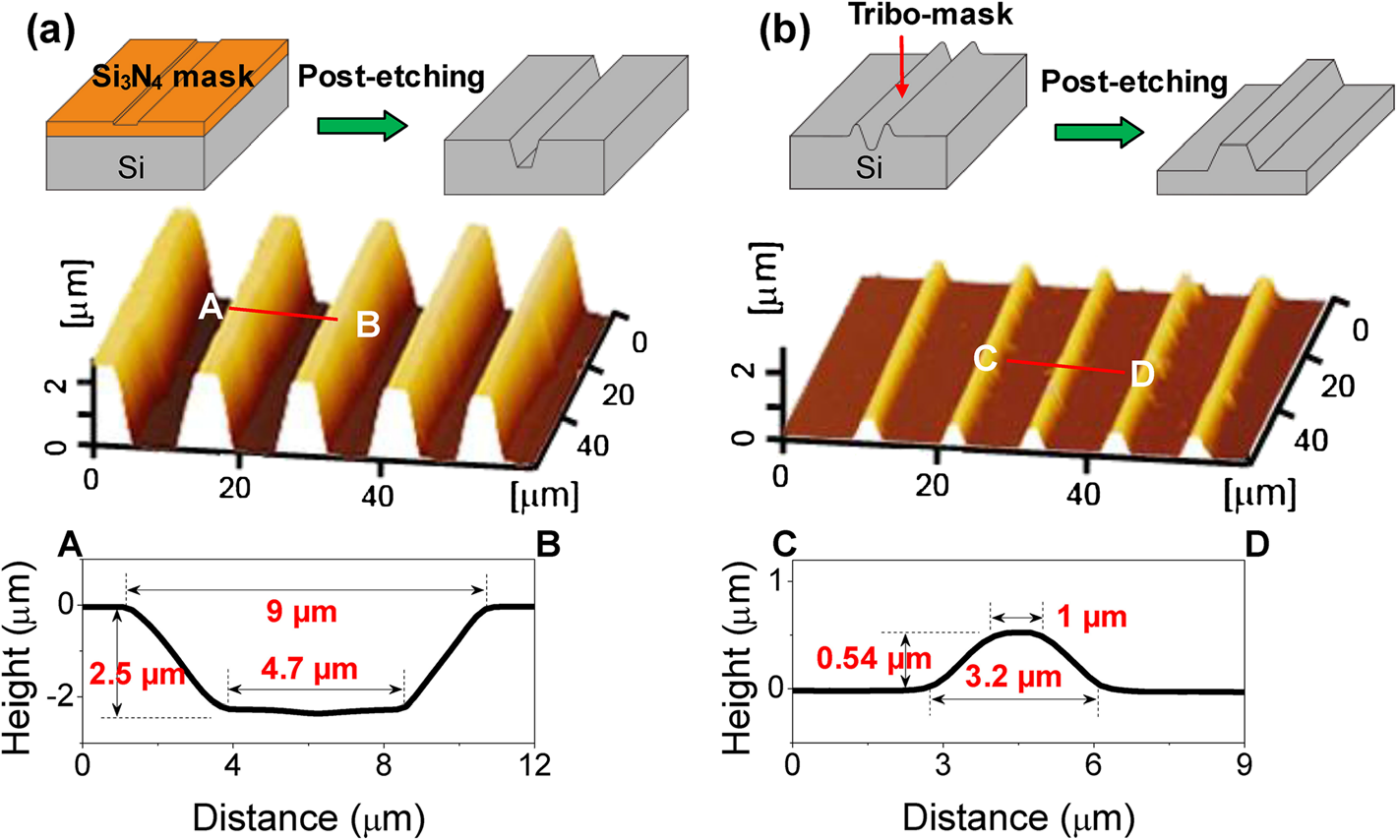
**Fabrication of line-array patterns by present method and the traditional friction-induced selective etching. (a)** Present method: line-array pattern with 2.5 μm in depth fabricated by scratching under *F*_n_ = 100 mN and post-etching in HF solution for 30 min and KOH solution for 4 h in sequence. **(b)** Traditional friction-induced selective etching: line-array pattern with 0.54 μm in height fabricated by scratching under *F*_n_ = 70 mN and post-etching in KOH solution for 1 h.

Compared to the traditional friction-induced selective etching, the present fabrication method can obtain deeper structure. Based on the high etching selectivity of Si(100)/Si_3_N_4_ in KOH solution, the maximum fabrication depth can reach several microns. As shown in Figure 7a, it was easy to produce a line-array pattern consisting of groove structures with a depth of 2.5 μm by using the present fabrication method. As a comparison, when fabricating nanostructure with the traditional friction-induced selective etching method, the amorphous layer generated by scratching played the mask role. The original silicon (on non-scratched area) was selectively etched by KOH solution so as to obtain a protrusive structure on the scanned area of the silicon surface, as shown in Figure 7b. Because of the low selectivity of Si(100)/tribo-mask, the maximum fabrication depth by the traditional friction-induced selective etching technique was only 0.54 μm. In addition, the present method can fabricate nanostructure with much lesser damage compared to the traditional friction-induced selective etching. When fabricating by the present method, the scratching was performed on the Si_3_N_4_ film. During the post-etching process, the scanned area was selectively etched. Hence, the fabricated patterns were almost composed of damage-free monocrystalline silicon structures. However, the structure fabricated by the traditional friction-induced selective etching may consist of a layer of amorphous silicon and deformed silicon on the surface, which may to some extent reduce the mechanical strength of the silicon structure. Therefore, considering the above advantages and potential application value, the present method will open up new opportunities for future nanofabrication fields.

## Conclusions

Based on the friction-induced selective etching of the Si_3_N_4_ mask, a new nanofabrication method was proposed to produce nanostructures on monocrystalline silicon. Experimental results suggest that HF solution can selectively etch the scratched Si_3_N_4_ mask and then provide the gap for KOH deep etching. The patterning structures with designed depth can be effectively fabricated on the target area by adjusting the scratching load and KOH etching period. Due to the excellent masking ability of the Si_3_N_4_ film, the maximum fabrication depth of 2.5 μm can be achieved. Compared to the traditional friction-induced selective etching, the advantage of the present method is to fabricate nanostructure with lesser damage and deeper depth. As a simple, flexible, and less destructive technique, the proposed method will provide new opportunities for Si-based nanofabrication.

## Abbreviations

AFM: atomic force microscope; IPA: isopropyl alcohol; LPCVD: low-pressure chemical vapor deposition; MEMS/NEMS: micro/nanoelectromechanical systems; RMS: root-mean-square; SEM: scanning electron microscope; SPM: scanning probe microscope; XPS: X-ray photoelectron spectroscopy.

## Competing interests

The authors declare that they have no competing interests.

## Authors' contributions

JG and XW finished the fabrication experiments and acquired the original data in this article. LQ and BY have made substantial contributions to the conception and design for this article. All the authors read and approved the final manuscript.
